# A density functional theory investigation of the electronic structure and spin moments of magnetite

**DOI:** 10.1088/1468-6996/15/4/044202

**Published:** 2014-08-05

**Authors:** Junghyun Noh, Osman I Osman, Saadullah G Aziz, Paul Winget, Jean-Luc Brédas

**Affiliations:** 1 School of Chemistry and Biochemistry and Center for Organic Photonics and Electronics, Georgia Institute of Technology, Atlanta, Georgia 30332-0400, USA; 2 School of Materials Science and Engineering, Georgia Institute of Technology, Atlanta, Georgia 30332-0245, USA; 3 Department of Chemistry, Faculty of Science, King Abdulaziz University, PO Box 80203, Jeddah 21589, Kingdom of Saudi Arabia

**Keywords:** DFT, spintronics, magnetite, ferrimagnetism, inverse spinel structure

## Abstract

We present the results of density functional theory (DFT) calculations on magnetite, Fe_3_O_4_, which has been recently considered as electrode in the emerging field of organic spintronics. Given the nature of the potential applications, we evaluated the magnetite room-temperature cubic 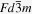 phase in terms of structural, electronic, and magnetic properties. We considered GGA (PBE), GGA + *U* (PBE + *U*), and range-separated hybrid (HSE06 and HSE(15%)) functionals. Calculations using HSE06 and HSE(15%) functionals underline the impact that inclusion of exact exchange has on the electronic structure. While the modulation of the band gap with exact exchange has been seen in numerous situations, the dramatic change in the valence band nature and states near the Fermi level has major implications for even a *qualitative* interpretation of the DFT results. We find that HSE06 leads to highly localized states below the Fermi level while HSE(15%) and PBE + *U* result in delocalized states around the Fermi level. The significant differences in local magnetic moments and atomic charges indicate that describing room-temperature bulk materials, surfaces and interfaces may require different functionals than their low-temperature counterparts.

## 1. Introduction

One of the most intriguing emerging areas in the field of organic electronics pertains to the development of organic spintronics [1–3]. Beyond applications that are well established already such as displays based on organic light-emitting diodes [4, 5] or much investigated such as organic solar cells [6], organic spintronics appears as a new avenue in which spin functionality is built into hybrid organic devices. In general, in such devices, a non-magnetic organic semiconductor is sandwiched between two ferromagnetic (FM) electrodes. There have been many materials considered so far as a source of spin injection, including ferromagnetic metals [7], dilute magnetic semiconductors [8], and Heusler alloys [9]. In particular, half-metallic ferro- (or ferri-) magnetic oxides can produce a very high magnetoresistive response. For instance, spin injection into organic semiconductors was first observed in a device consisting of sexithienyl deposited on La_0.7_Sr_0.3_MnO_3_ (LSMO) [10]. In an effort to improve on the electrode characteristics beyond LSMO, magnetite (Fe_3_O_4_) has been proposed as a result of its high Curie temperature (∼850 K), the possibility of formation of high-quality thin films with well-defined magnetic anisotropy, and the less reactive nature of the surface leading to weaker hybridization effects compared to FM metals [11, 12]. Importantly, Fe_3_O_4_ shows an exceptionally large spin polarization at the Fermi level at room temperature.

The chemical interactions between organic molecules and electrodes play a significant role in the electronic and magnetic structure of the interface. For example, it was demonstrated using x-ray absorption spectroscopy and x-ray magnetic circular dichroism that electronic interactions between C_60_ 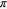
(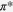) and Fe_3_O_4_ 3*d* states lead to interfacial electronic states of importance in the spin injection mechanism [12]. Here, as a first step towards ultimately exploring organic-magnetite interfaces, we have chosen to focus on the theoretical description of the electronic and magnetic properties of magnetite itself.

**Figure 1.**
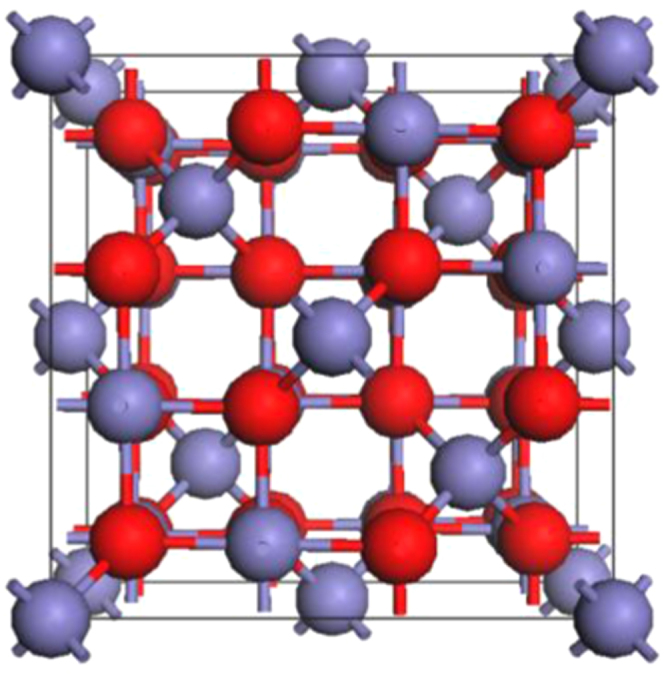
The crystal structure of magnetite (Fe_3_O_4_) in its cubic 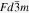 phase. Tetrahedral-site Fe atoms (8/unit cell) and octahedral-site Fe atoms (16/unit cell) are in blue, and oxygen atoms (32/unit cell) are in red.

Magnetite has a cubic inverse spinel structure (space group 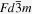) at room temperature with 8 formula units (f.u.) in the conventional unit cell as shown in figure 1. Its chemical formula, often written as [Fe^3+^]_A_ [Fe^3+^,Fe^2+^]_B_ O_4_, indicates that the tetrahedral sites denoted as A are occupied by ferric ions while octahedral sites denoted as B contain an equal number of ferric and ferrous ions. In magnetite, the tetrahedral and octahedral sites form two magnetic sublattices with the spin moments on the A sublattice antiparallel to those on the B sublattice. The proposed electronic structure of the octahedral Fe^2+^ cations corresponds to a situation where an extra electron resides in the lowest unoccupied *t*_2g_ orbital located at the Fermi level. Such an occupation then would give rise to the 100% spin-polarized charge carriers desired for spintronic applications [13, 14].

Magnetite displays an electrical conductivity as high as 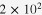 S cm^−1^ in the thermodynamic standard state; however, it undergoes a Verwey phase transition [15] with a clear opening of the optical gap, ∼0.14 eV at 121 K [16], a temperature below which the electronic conductivity abruptly decreases by two orders of magnitude. This quasi metal–to–insulator transition has been explained by restricted electron hopping between Fe^2+^ and Fe^3+^ ions in the octahedrally coordinated positions due to charge ordering below the transition temperature [17, 18]. In addition to the increase in electrical resistivity and changes in magnetization and heat capacity, this transition is also accompanied by a structural distortion from the room-temperature cubic system; orthorhombic [19], monoclinic [20], and triclinic [21] unit cells have been observed at low temperatures.

Although magnetite has been extensively investigated in past decades, most of the studies have focused on the low-temperature monoclinic *Cc* phase (i.e., on the charge-ordered structure below the Verwey transition temperature), while there are only few reports on the high-temperature cubic 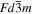 phase. However, it is the structure at ambient temperature that is relevant in the actual operating conditions for many of the applications of magnetite [15]; therefore, a detailed understanding of the physical and chemical nature of the cubic system is clearly needed.

Despite the fact that standard density functional theory (DFT) calculations provide overall a reasonable description of the structural properties and magnetic ground state of iron oxides, they often fail to provide an accurate determination of the electronic structure of hematite (*α*-Fe_2_O_3_) [16, 17], magnetite [18], and goethite (*α*-FeOOH) [19]. One of the issues is that owing to the strong correlation effects among Fe 3*d* electrons which lead to a splitting of the *d* bands, iron oxides and oxyhydroxides can be calculated to be either semiconducting or metallic depending on the relative positions of the oxygen 2*p* and iron 3*d* orbitals in the valence bands. Another challenge for these theoretical methods is the ability to quantify the degree of localization of the charge on the B sublattice, as this can dictate key features of the electronic structure around the Fermi level [20]. One method that improves on these limitations relies on the modification of the intra-atomic Coulomb interactions using the DFT + Hubbard *U* (DFT + *U*) approach [21]. More recently, hybrid exchange-correlation (XC) functionals including a fraction of the Hartree–Fock (HF) exchange have been applied, and have been particularly successful in describing the ground-state properties of a wide class of transition metal oxides [22]. While the influence of various XC methods on the electronic structure of the organic layers in organic–inorganic heterostructures has been extensively studied, a detailed description of the impact methodological impact on the electronic structure of metal oxides is still lacking [23].

Here, our goal is to present a comprehensive DFT investigation of cubic-phase Fe_3_O_4_ using various treatments for XC functionals using the GGA, GGA + *U*, and range-separated hybrid functional approaches. Our work underlines that the calculated band gap energies but as well the very nature of the valence band strongly depend on the choice of functional. This understanding represents an important step prior to extending our calculations to Fe_3_O_4_ surfaces and their interfaces with organic layers.

## 2. Computational methodology

First-principles calculations have been performed using spin-polarized DFT as implemented in the Vienna *ab initio* Simulation Package [24, 25]. The ionic potentials are described by the projector augmented wave pseudopotential [26] with valence configurations of 3*d*^6^4*s*^1^ and 2*s*^2^2*p*^4^ for Fe and O atoms, respectively. In the course of optimization of the crystal structure, the ion positions were allowed to relax by applying a Gaussian-smearing approach with 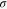 = 0.05 eV until the Hellmann–Feynman forces were less than 0.02 eV Å^−1^ and energy convergence was reached within 10^−4^ eV atom^−1^. Atomic charges were estimated within the Bader scheme [27, 28].

We used the GGA XC functional of Perdew, Burke, and Ernzerhof (PBE) [29, 30] for both DFT and DFT + *U* approaches. The PBE + *U* method used here is a simplified rotationally invariant formulation by Dudarev *et al* [31]. Where the on-site Coulomb parameter, *U*, and exchange parameter, *J*, are combined into a single parameter, *U*_eff_ ≡ *U* – *J*. We chose *U*_eff_ = 4 eV for the strongly correlated Fe 3*d* electrons based on the previous computational estimate of Zhang and Satpathy [13]. This value has been shown to be valid for other iron oxides as it gives accurate lattice constants, magnetic moments, bulk modulus, and band gap energies for *α*-Fe_2_O_3_ [32] and FeOOH [33]. The Brillouin zone integration was performed using Monkhorst-Pack grids with a 5 × 5 × 5 mesh for relaxation of bulk structures with cut-off energy of 550 eV.

We also considered a range-separated hybrid functional following the scheme proposed by Heyd, Scuseria, and Ernzerhof (HSE) [34, 35], which separates the exchange energy into short-range (SR) and long-range (LR) components. The SR exchange contains both HF and PBE terms, while the LR exchange interactions are composed of PBE exchange only; the correlation part is PBE in all regions. The resulting functional can be written as:

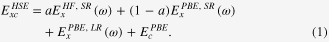
Here, 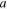 indicates a HF exchange mixing coefficient and 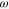 is an adjustable parameter that defines the partitioning between the SR and the LR. The value, 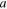 = 0.25, is the portion of exact exchange chosen from perturbation theory. We present results using 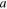 = 0.25 and 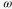 = 0.11 bohr^−1^, i.e., the HSE06 functional, as it has been shown to accurately predict enthalpies of formation, ionization potentials, and electron affinities for molecules as well as lattice constants and band gaps of solids in general [36]. Considering the fact that the optimal amount of HF exchange is system dependent for hybrid functionals [37], we also present results obtained using a reduced value of 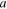, 0.15, referred as HSE(15%), based on previous work using the hybrid B3LYP functional [38]. All range-separated calculations were performed using 3 × 3 × 3 *k*-point meshes and a 500 eV cutoff on the basis of geometries optimized with PBE + *U*.

## 3. Results and discussion

The Fe_3_O_4_ unit cell containing 24 Fe and 32 O atoms was fully relaxed at the PBE and PBE + *U* levels *while preserving cubic symmetry*. As presented in table 1, the PBE lattice constant is 8.387 Å, nearly identical to the experimental value, 8.396 Å [15]; PBE + *U* slightly overestimates the experimental value by 1% (8.488 Å). In addition to the minor difference in lattice constant, the bond lengths between Fe and surrounding O atoms are calculated to be longer by 0.02–0.03 Å upon consideration of on-site Coulomb interactions among Fe 3*d* electrons, which effectively decreases the charge density in the Fe–O bonds. In terms of the energy of formation per O atom, i.e., equating the internal energy to the Gibbs free energy, the PBE + *U* calculations result in a value much closer to the experimental free energy of formation, of −3.12 eV versus −2.89 eV per O atom at low temperature [39] than PBE, which underestimates the value by 20%.

**Table 1.** Lattice constant, interatomic distances and energy of formation (per O) of cubic Fe_3_O_4_ calculated using PBE and PBE + *U*. The experimental lattice constant and Gibbs free energy of formation is listed for comparison.

	PBE	PBE + *U*	Exp.
Lattice constant (Å)	8.387	8.488	8.396[15]
*d*(Fe_tet_-O) (Å)	1.88	1.9	—
*d*(Fe_oct_-O) (Å)	2.06	2.09	—
*ΔE*_*f*_ (eV)	−2.31	−3.12	−2.89^a^(−2.91^b^[39])

a *ΔG*_*f*_ (0 K).

b *ΔG*_*f*_ (298 K).

In cubic-phase magnetite at room temperature, electron hopping occurs between the Fe^2+^ and Fe^3+^ sites of the mixed-valence octahedral plane, resulting in an average oxidation level of Fe^2.5+^ per occupied site and a magnetic moment of 4 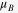 per Fe_3_O_4_ formula unit. While both PBE and PBE + *U* calculations provide the same net magnetic moment of 4 *μ*_*B*_/f.u., the descriptions of the local magnetic moments and Bader charges for the Fe_oct_, Fe_tet_, and O atoms vary with the consideration of the effective Coulomb interaction, as shown in table 2. The magnetic moment of the tetrahedral Fe atom is calculated with PBE to be 3.47 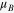, which is much smaller than the value of 3.82 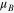 for the experimentally measured one [40]. This points to a strong hybridization among the 3*d* orbitals of Fe_tet_ with the surrounding oxygen atoms. Adding the modified Coulomb repulsion, *U*_eff_, in the PBE + *U* calculations improves the agreement with experimental data by increasing the magnetic moment by 0.6 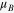 and the electron charge by 0.18*e* compared to the values in PBE. Similar trends are obtained for Fe_oct_ atom: the magnetic moment is 0.4 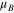 lower with PBE than PBE + *U* and atomic charges are smaller by 0.12*e*.

**Table 2.** Magnetic moment 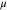 and Bader charge, *q*, of each element of bulk Fe_3_O_4_ calculated using PBE, PBE + *U*, HSE06, and HSE(15%). The HSE06, and HSE(15%) calculations are based on the PBE + *U*-optimized geometry. The minimum and maximum values of the magnetic moment are tabulated for the four Fe_oct_ ions.

	PBE		PBE + *U*		HSE06		HSE (15%)	
	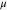 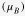	q (*e*)	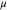 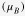	q (*e*)	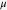 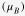	q (*e*)	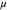 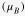	q (*e*)
Fe_oct_	3.55–3.58	+1.60	3.96–3.98	+1.72	3.49–4.37	1.82	3.82–3.85	1.71
Fe_tet_	3.47	+1.68	4.09	+1.86	4.06	1.99	3.92	1.89
O	0.08	−1.22	0.03	−1.32	0.03	−1.40	0.06	−1.33

**Figure 2.**
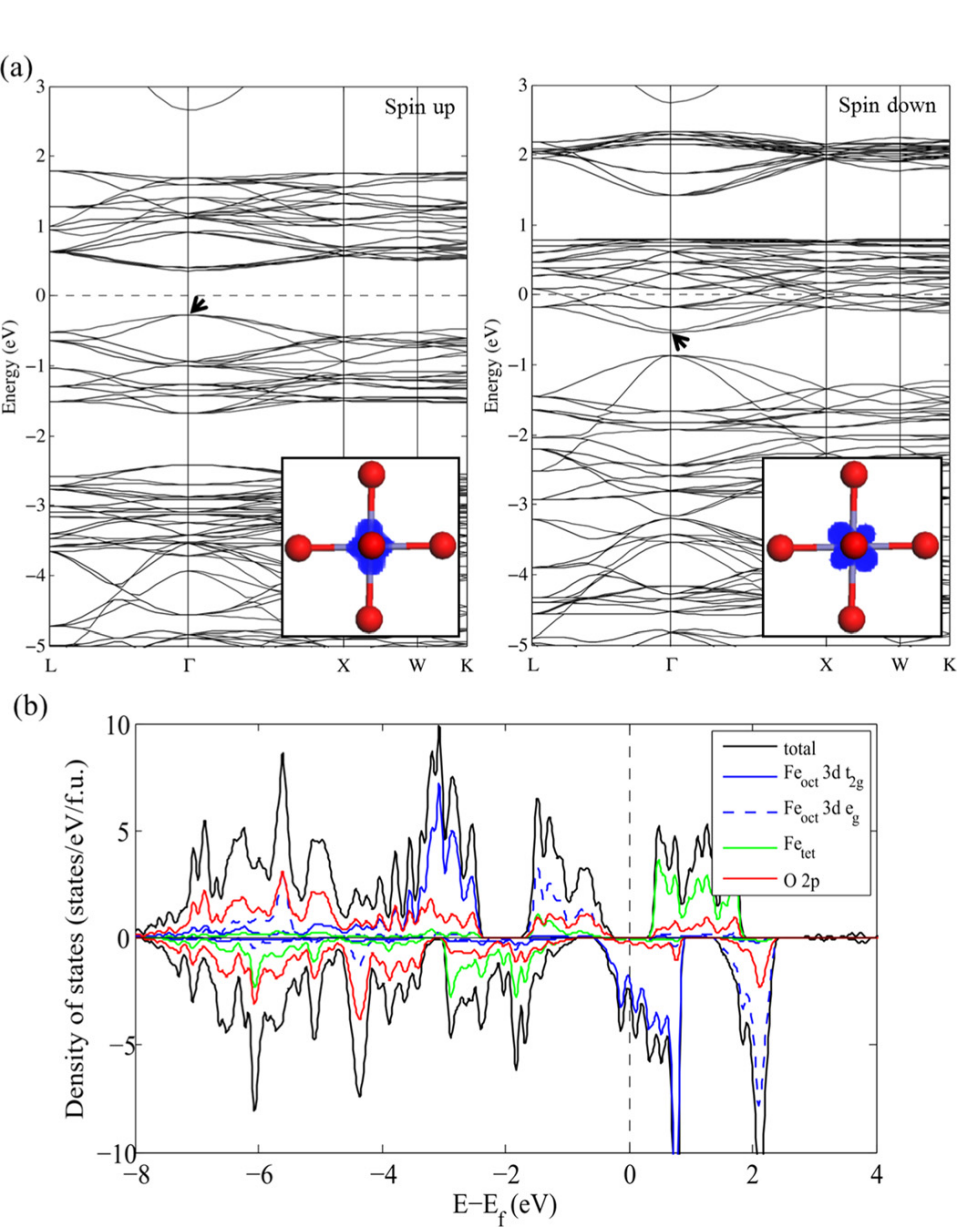
PBE electronic band structure (a) and density of states (b) of Fe_3_O_4_ in the 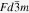 unit cell. The partial charge density at valence band maximum or conduction band minimum (VBM or CBM, as indicated with an arrow) is shown in the inset of (a). The blue, green, red and black lines in (b) represent surface Fe_oct_ 3*d*, Fe_tet_ 3*d*, O 2*p* states, and total states respectively. The Fermi level (=zero of energy, see text) is indicated with a dashed line.

**Figure 3.**
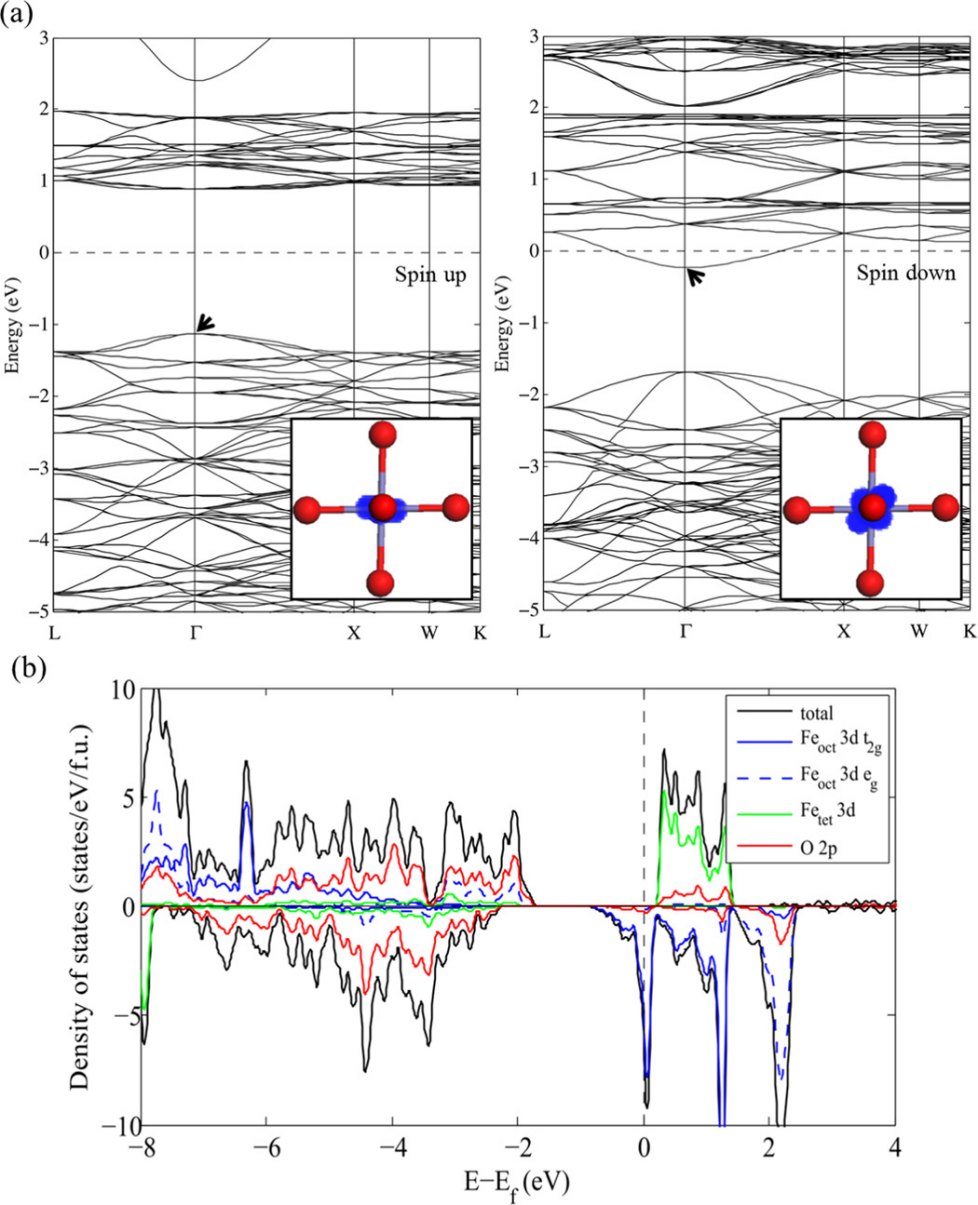
PBE + *U* electronic band structure (a) and density of states (b) of Fe_3_O_4_ in the 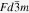 unit cell. The partial charge density at VBM or CBM (as indicated with an arrow) is shown in the inset of (a). The blue, green, red and black lines in (b) represent surface Fe_oct_ 3*d*, Fe_tet_ 3*d*, O 2*p* states, and total states respectively. The Fermi level (=zero of energy, see text) is indicated with a dashed line.

The band structure and partial density of states (PDOS) projected on the Fe_oct_, Fe_tet_ and O sites were obtained from the PBE and PBE + *U* calculations for the 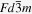 unit cell as shown in figures 2 and 3, respectively. For consistency between the metallic and semiconducting systems, the Fermi energy (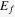) is taken here as the highest occupied energy level of the system. Both the PBE and PBE + *U* results indicate that cubic Fe_3_O_4_ is a half-metallic oxide where the majority spin band exhibits insulating or semiconducting behavior whereas the minority spin band shows metallic behavior. However, the specific details of the electronic structure are significantly different when using the two methodologies.

As shown in figure 2(a), using the PBE functional results in an electronic structure with a direct band gap of 0.6 eV at the Γ point in the majority spin band structure. The majority spin valence band maximum consists of Fe 3*d e*_g_ orbitals and O 2*p* orbitals in nearly equal proportions, while the conduction bands are mainly comprised of 3*d* states from tetrahedral Fe atoms. As indicated in figure 2(b), localized 3*d* states of Fe_oct_ atoms are present between −2.5 eV and −3.8 eV and delocalized oxygen 2*p* orbitals appear well below 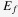. In the minority spin band structure, the results show that the *t*_2g_ states from Fe_oct_ ions dominate the DOS around 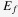, which is consistent with previous studies [41]. The PBE functional locates the valence band at low binding energy, resulting in the majority valence band and the minority conduction band virtually overlapped at 0.3 eV below the Fermi energy. The PDOS does not replicate the observation of band discontinuities at the Fe 3*p* → 3*d* resonance photon energy for the high-lying Fe 3*d*-derived bands [42].

Figure 3 shows that the PBE + *U* describes the semiconducting nature in the majority spin state of Fe_3_O_4_ with an increased band gap, 2.1 eV. At the valence band maximum, the contribution of O 2*p* orbitals increases from 51% to 82% and there is no longer a localized Fe_oct_ 3*d* band below the hybridized states. This is attributed to the Fe 3*d* states originally lying close to Fermi level now shifted to higher binding energy due to on-site Coulomb interaction among Fe 3*d* electrons. In contrast to the results from PBE, PBE + *U* shows the valence band extending from 2 to 9 eV, which is consistent with the presence of O 2*p*-derived states in the 3–8 eV range and Fe 3*d*-derived states at 4 eV below the 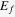 in a previous photoemission study [43]. The minority spin structure obtained from PBE + *U* has a large gap of 1.9 eV between Fe_oct_ 3*d t*_2g_ states and O 2*p* hybridized states at *Γ*, which gives rise to an overall band discontinuity between the majority and minority spin channel from −0.3 eV to −1.1 eV. The minority spin Fe_oct_ 3*d t*_2g_ states at the Fermi level are slightly narrower than when using the PBE functional. Thus, at the PBE + *U* level, states separated from the majority spin Fe_tet_ states rather than overlapping them as in PBE. These results show nearly 100% spin polarization, which is in fair agreement with previous calculations [13] and spin-polarized photoemission experiments [44, 45].

**Figure 4.**
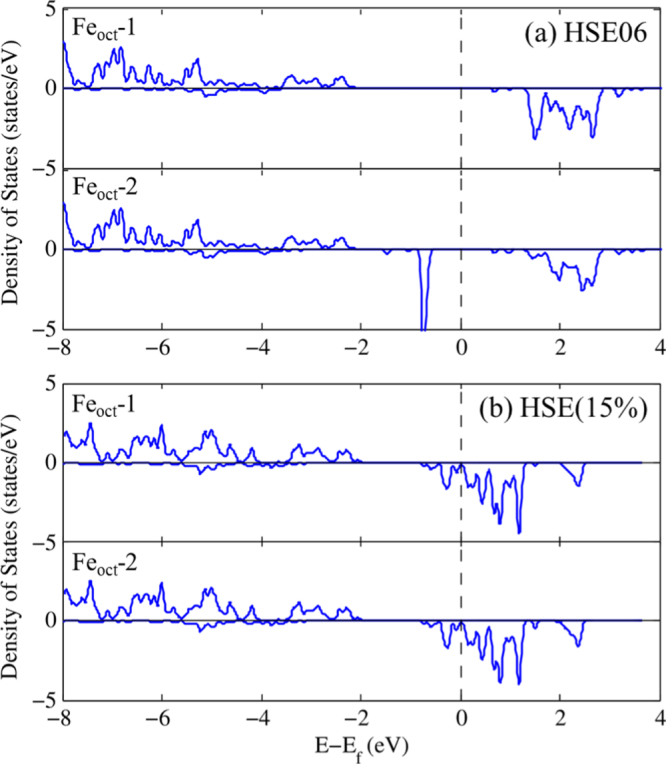
Density of states for Fe_oct_ atoms in two different positions: Fe_oct_-1 (0.625, 0.625, 0.625) and Fe_oct_-2 (0.625, 0.125, 0.125) in the cubic 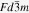 phase using the (a) HSE06 and (b) HSE(15%) functionals.

To put our results in a broader perspective, we also performed range-separated hybrid HSE06 and HSE(15%) calculations which correct for the self-interaction error by partial inclusion of HF exchange in the short range. We note that while the PBE + *U* functional produces a stable structure in the cubic point group, previous optimizations at the hybrid-DFT (B3LYP) level leads to a structure with no symmetry [38]. Thus, we here utilized the optimized PBE + *U* structure for these further calculations, which is similar in spirit to previous studies where the lattice parameters were fixed at experimental values. As presented in table 2, HSE06 leads to a localization of the Fe 3*d* electrons, and significant reduction of magnetic moments in Fe_oct_ atoms where the values range from 3.49 to 4.37 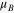; this is in contrast with the PBE and PBE + *U* results that are more uniform, the maximum difference being 0.03 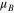. In order to confirm this charge localization presented by the HSE06 functional, the PDOS corresponding to two distinct sites of Fe_oct_ ions with fractional coordinates (Fe_oct_-1: 0.625, 0.625, 0.625; and Fe_oct_-2: 0.625, 0.125, 0.125) are plotted in figure 4(a). It is clear that the electronic structures around Fe_oct_-1 and Fe_oct_-2 are different: there is no state at 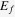 for Fe_oct_-1 (Fe^3+^) while a distinctive peak below 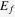 is present for Fe_oct_-2 (Fe^2+^). We note that HSE06 calculations incorporating 25% of exact exchange in the short range fail to describe the observed room-temperature symmetrical charge distribution over Fe_oct_ atoms of the unit cell in spite of symmetry constraints imposed by consideration of a cubic structure. This PDOS is similar to the one obtained for Fe_3_O_4_ in a lower symmetry 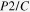 unit cell in the work of Rowan *et al* [38]. Distortions of Fe B site octahedra can be caused by symmetry breaking due to charge ordering [38], which can also be characterized by disproportionation of magnetic moments among Fe_oct_ atoms and opening of a *d*–*d* optical band gap [46]. On the other hand, when using a smaller fraction of HF exchange, 15%, in the short range, the two distinct Fe_oct_ atoms retain the same electronic structure, as shown in figure 4(b). The magnetic moments and atomic charges deduced from this electronic structure are similar to the PBE + *U* values although the magnetic moments of Fe atoms are slightly smaller.

**Figure 5.**
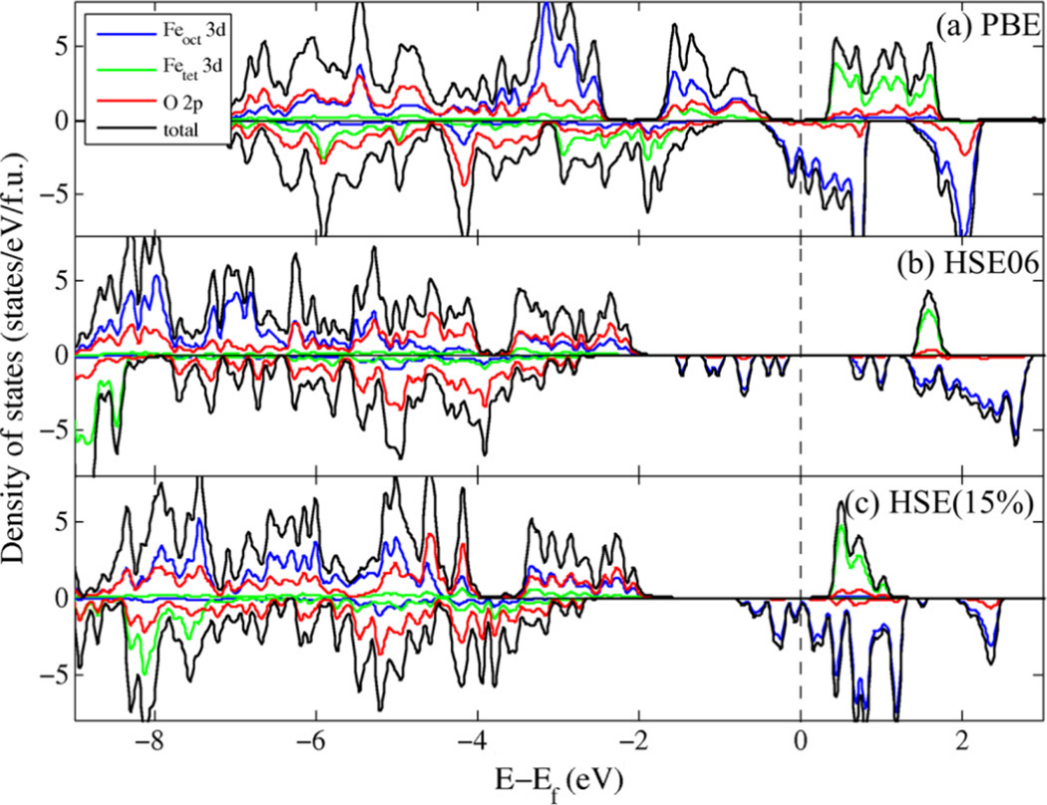
Projected density of states of bulk Fe_3_O_4_ with (a) PBE, (b) HSE06, (c) HSE(15%) functional. The blue, green, red and black lines represent surface Fe_oct_ 3*d*, Fe_tet_ 3*d*, O 2*p* states and total states respectively. The Fermi level is indicated by a dashed line.

Figure 5 shows the total DOS and PDOS projected on the Fe_oct_, Fe_tet_ and O sites with PBE, HSE06, and HSE(15%) using the PBE + *U*-optimized structure. (We note that we used a different crystal structure, the PBE + *U* structure, in figure 5(a), while we used the optimal PBE structure in figure 2(b).) In spite of some similarities in the description of electronic structures of bulk magnetite, there is a noticeable difference in the band gap energies and the nature of the Fe 3*d* orbitals near the Fermi level. When comparing figures 5(a) and 2(b), the electronic structure is nearly identical, suggesting that the resulting electronic structure is insensitive to the specific variations in geometry.

In range-separated hybrid-DFT calculations, the majority band gap energies decrease from 3.4 to 2.2 eV along with the stabilization of the conduction band as the fraction of HF exchange in the functional is reduced from 25% to 15%, as shown in figures 5(b) and (c). This is consistent with previous B3LYP results where the band gap in magnetite is extremely sensitive to 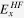, as the inclusion of 20% exact exchange gives a band gap of 0.87 eV, which is reduced to 0.32 eV when the fraction of exchange is reduced to 15% [38]. The nature of the valence band maximum at the Γ point changes upon reduction of the fraction of exact exchange; the oxygen *p* orbital contribution decreases slightly from 68% to 62% in the HSE(15%) calculation. More significantly, HSE06 shows a distinctive series of Fe_oct_ localized 3*d* states right below 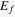 while in HSE(15%) these states are dispersed around 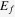. When combined with the disproportionation of magnetic moments among Fe_oct_ atoms, this difference indicates that these ions can be represented as distinct Fe^2+^ and Fe^3+^ ions when using HSE06 while the HSE(15%) results represent a delocalized system with a series of Fe^2.5+^ ions. The PBE + *U* PDOS shown in figure 3(b) most closely resembles the HSE(15%) results, with the Fe_oct_ 3*d* states dispersed around the Fermi level and the majority spin Fe_tet_ states slightly above the Fermi level.

## 4. Conclusions

We have presented DFT-GGA, GGA + *U* and range-separated hybrid-DFT calculations of magnetite, Fe_3_O_4_. Given its potential application as electrode in organic spintronics, we have considered the magnetite room-temperature cubic 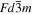 phase in terms of structural, electronic, and magnetic properties. We find that structural relaxation with the PBE and PBE + *U* functionals show slight differences in the lattice constant, while ionic relaxation using the HSE06 functional leads to symmetry broken structure. Both PBE and PBE + *U* describe cubic Fe_3_O_4_ as a half-metallic oxide, however, there are noticeable differences concerning the band gap energies and the nature of valence band maximum on the semiconducting majority spin channel. The results using HSE06 and HSE(15%) functionals indicate that inclusion of exact exchange has a significant impact on the electronic structure as HSE06 predicts highly localized states below the Fermi level while HSE(15%) and PBE + *U* predict delocalized states around the Fermi level. There are significant discrepancies in the formation energy, local magnetic moments, and atomic charges depending on the functional used, with PBE + *U* and HSE(15%) providing a better overall agreement with high-temperature experimental data. Given the significant computational savings provided by PBE + *U*, this methodology opens the way for accurate calculations on large unit cells. While the modulation of the band gap with exact exchange has been seen in numerous situations, the dramatic change in the valence band nature and states near the Fermi level has significant implications for choice of DFT functional for future calculations. The use of HSE06, which predicts the symmetry broken structure below Verwey transition temperature, may not be applicable for understanding the room-temperature surfaces and interfaces required to optimize devices and magnetite-catalyzed reactions.
